# Short‐ and longer‐term impacts of Child Friendly Space Interventions in Rwamwanja Refugee Settlement, Uganda

**DOI:** 10.1111/jcpp.13069

**Published:** 2019-05-20

**Authors:** Janna Metzler, Karin Diaconu, Sabrina Hermosilla, Robert Kaijuka, George Ebulu, Kevin Savage, Alastair Ager

**Affiliations:** ^1^ Department of Population and Family Health Mailman School of Public Health Columbia University New York NY USA; ^2^ Institute for Global Health and Development Queen Margaret University Edinburgh Scotland UK; ^3^ New York State Psychiatric Institute Columbia University New York NY USA; ^4^ World Vision Uganda Kampala Uganda; ^5^ Humanitarian and Emergency Affairs World Vision International Geneva Switzerland

**Keywords:** Humanitarian crisis, refugees, protection, psychosocial support, impact, longitudinal

## Abstract

**Background:**

The establishment of Child Friendly Spaces (CFSs) has become a widespread intervention targeting protection and support for displaced children in humanitarian contexts. There is a lack of evidence of impact of these interventions with respect to both short‐term outcomes and longer‐term developmental trajectories.

**Methods:**

We collected data from caregivers of Congolese refugee children residing in Rwamwanja Refugee Settlement at three timepoints. To assess short‐term impact of CFSs, we compared indicators assessed shortly after refugees’ arrival (baseline, T1) and endline (T2, three to six months after CFS implementation) amongst 430 CFS attenders and 161 nonattenders. Follow‐up assessments after the end of CFS programming were conducted 18 months post‐baseline (T3) with caregivers of 249 previous CFS attenders and 77 CFS nonattenders.

**Results:**

In the short‐term, attendance at CFSs was associated with better maintenance of psychosocial well‐being (PSWB; β = 2.093, *p* < .001, Cohen's *d* = .347) and greater increases in developmental assets (β = 2.517, *p* < .001, Cohen's *d* = .231), with significantly stronger impacts for girls. CFS interventions meeting higher programing quality criteria were associated with greater impact on both PSWB and development assets (β = 2.603 vs. β = 1.793 and β = 2.942 vs. β = 2.337 for attenders at higher and lower‐quality CFSs c.f. nonattenders, respectively). Amongst boys, benefits of program attendance were only indicated for those attending higher‐quality CFS (β = 2.084, *p* = .006 for PSWB). At follow‐up, however, there were no discernable impacts of prior CFS attendance on any measures. Age and school attendance were the only characteristics that predicted an outcome – developmental assets – at follow‐up.

**Conclusions:**

Attendance at CFSs – particularly involving higher‐quality programming – supported children's well‐being and development. However, sustained impact beyond active CFS programming was not demonstrated. Intervention goals and strategies in humanitarian contexts need to address the challenge of connecting children to other resources to facilitate developmental progress in conditions of protracted displacement.

## Introduction

Forced displacement currently impacts the lives of more than 65 million people globally (UNHCR, 2017). Children comprise roughly half of this population (UNHCR, 2017). Child refugees fleeing conflict settings are known to face many threats to their safety and well‐being. In addition to direct exposure to physical and sexual violence, children are vulnerable to military training and recruitment, separation from their families, early marriage, and child labor (Ager, Blake, Stark, & Daniel, 2011; Global Protection Cluster Child Protection Working Group, 2015). Further, there is extensive literature documenting the potential sequelae of forced displacement from situations of conflict, which indicates an elevated risk for post‐traumatic stress disorders and other severe mental conditions, as well as for common mental disorders and behavioral problems (Reed, Fazel, Jones, Panter‐Brick, & Stein, 2011; Tol, Song, & Jordans, 2013).

Strategies for addressing these challenges to protection and well‐being have become an increasing focus of research (Bangpan, Dickson, Felix, & Chiumento, 2017; Brown, de Graaff, Annan, & Betancourt, 2016). With a growing emphasis on the role of social ecologies in shaping the experience of children (Betancourt et al., 2011; Masten & Narayan, 2012; Miller & Rasmussen, 2010), studies have considered not only the effectiveness of specialist clinical interventions but also of nonspecialist treatment and prevention programs (Betancourt, Meyers‐Ohki, Charrow, & Tol, 2013; Jordans, Tol, Komproe, & de Jong, 2009). However, to date, relatively little attention has been paid to assessing the impact of the forms of structured programming for children routinely deployed by humanitarian agencies in the context of crisis response (Bangpan et al., 2017; Blanchet et al., 2017).

Child Friendly Spaces (CFSs) are increasingly adopted as the presumptive intervention strategy by humanitarian agencies to promote the mental health and psychosocial well‐being (PSWB) of children in crisis contexts. They are seen as a safe environment in which children may resume some sense of normalcy (Ager, Metzler, Vojta, & Savage, 2013; UNICEF, 2009; Wessells & Kostelny, 2013). CFSs are attractive to practitioners wanting a scalable program with adaptable and diverse activities that is readily deployable in challenging contexts (Kostelny & Wessells, 2008, 2013; Madfis, Martyris, & Triplehorn, 2010; Metzler et al., 2013; Save the Children, 2008, Save the Children, 2009; Save the Children Sweden, 2010; UNICEF, 2009; World Vision International, 2006). The aims of CFS programming are generally threefold. First, CFSs seek to provide and promote children's emotional well‐being, social well‐being, and in some cases, support the acquisition of skills and knowledge (Global Protection Cluster [GPC], Global Education Cluster [GEC], International Network for Education in Emergencies [INEE], Inter‐agency Standing Committee [IASC], 2011). Second, CFSs serve as a mechanism for protecting children from abuse, exploitation, and/or violence (GPC et al., 2011). Third, CFSs may aim to mobilize communities around these first two aims to further strengthen community mechanisms that support, protect, and care for children (GPC et al., 2011). Although widely varied in operation, the core elements of CFSs are typically: establishment of a dedicated, safe physical environment (generally with facilities for play and/or learning); the recruitment, training, and support of facilitators (usually from the community from which children are themselves drawn); and the provision of structured activities in a regular timetable of activities utilizing the above resources (Ager et al., 2013; Wessells & Kostelny, 2013).

Despite widespread consensus of the intervention's key objectives and subsequent global adoption of its use in emergency settings, little robust evidence exists related to programmatic outcomes and impacts (Ager et al., 2013; GPC et al., 2011). In a systematic review of published and gray literature, only ten studies were found that met inclusion criteria, with most displaying major design weaknesses that restricted the ability to robustly confirm positive change over time or attribute such change to programmatic efforts (Ager et al., 2013). Broader reviews of the evidence base for humanitarian interventions addressing the mental health and PSWB of displaced populations echo these findings (Blanchet et al., 2017; Tol, Barbui et al., 2011; Tol, Patel et al., 2011). Reviews particularly emphasized the need for more rigorous, mixed methods research on the effectiveness of widely used, group‐based psychosocial interventions, particularly those aimed at children and adolescents, such as CFS.

### The current study

The current study builds upon an initiative by World Vision International and Columbia University, working with Save the Children, United Nations Children’s Fund (UNICEF) and other members of the Global Protection Cluster Child Protection Working Group, to explore the protective and restorative outcomes and impacts of CFSs in various emergency contexts (World Vision and Columbia University, 2015). Although CFSs are recognized as short‐term, emergency interventions seeking immediate impact on children's well‐being, their rationale frequently also implies the benefits of intervention protecting the longer‐term development trajectories of children. This study is the first rigorous analysis of both short‐ and longer‐term impacts of CFSs, following the progress of children from their first arrival as refugees in Uganda to some eighteen months later.

## Methods

### Setting

Early 2012 ushered in the development of a new fighting force, the M23 Movement, and its infiltration of the North Kivu province of the Democratic Republic of Congo (DRC; IRIN, 2012). By late 2012, Uganda was steward to over 130,000 asylum‐seekers and refugees, many of which came to reside in settlement camps in Western Uganda (UNHCR, 2012a). The Office of the Prime Minister (OPM) in coordination with UNHCR and WFP has met this conflict with a longer‐term strategy to promote farming as a livelihood amongst settlement members to help ease the future burden for external food assistance and strengthen local markets (UNHCR, 2014).

Bordering the Katonga Game Reserve in the Kamwenge District of southwestern Uganda, Rwamwanja Refugee Settlement covers over 40 square miles of woody savanna with patches of dense thicket, rolling hills, and seasonal ponds (International Union for Conservation of Nature, United Nations Environment Programme & Rwenzo‐Green Associates, 2013). The settlement was re‐opened in April 2012 to support the swelling conflict, and by the start of the current study in late September 2012, over 20,000 refugees had taken up residence (UNHCR, 2012b). By the study's conclusion, the settlement had reached its capacity with over 50,000 refugees in residence (UNHCR, 2014). Early in its development, the settlement was demarcated into *villages* that varied in population size, each being divided into further subunits named *nyumba kumi*, derived from a local Congolese community structure normally constituting 10 households, but incorporating as many as 25 in this setting.

### Intervention

In coordinated operations, World Vision Uganda and Save the Children in Uganda phased implementation of twenty CFSs across the settlement. Construction of these CFSs, locally referenced as *obebes*, began in August 2012 with phased construction and commencement of program activities continuing until November 2012. At the start of the study in late September 2012, discussions with both program teams were held to determine intended implementation strategies for remaining CFSs and confirm timelines for key evaluation and program activities. Five villages where settlement was ongoing were identified as the catchment areas for a total of eight CFSs due to become operational within the following 6 months, which thus formed the cohort of interventions addressed by the study. The physical attributes of all CFSs were similar and consisted of a tented activity area, latrines, a store, and a variety of playground equipment. CFS activities were available for children 6–12 years old, 5 days per week, led by trained facilitators drawn from the settlement area. Four‐hour sessions included a range of psychosocial activities, such as traditional song and dance, art, organized sports and unstructured free play, and educational components, such as basic literacy and numeracy skills in the local dialect and English languages. Supervising staff led initial community meetings with adults and children introducing the CFS, monitored CFS activities and supported sensitization meetings for children, youth, and caregivers on child rights and protection. Enrollments at any one CFS ranged from 65 to 651 children. The intervention was explicitly funded as an emergency program to support settlement of children within the camp during its first year of operation.

Intervention quality was assessed during a site visit using a 10‐item CFS Quality Standards Checklist, assessing standards regarding child protection protocols, activities, safety of equipment, record‐keeping, and planning drawn from a broader Quality Standards for Children's Activities and CFS Programmes Assessment (World Vision International, 2006). Items were worded as objectively verifiable standards (e.g. ‘no broken playground equipment in evidence’, ‘children's work on display’, ‘first aid kit available and stocked’). Data were combined from two site visits (by members of the research team) and a full quality standards assessment by a child protection specialist of one of the implementing agencies. With a bimodal distribution of scoring, a score of 60% served to define the dichotomous threshold between the four CFS operating with ‘higher quality’ and the four operating with ‘lower quality’.

### Design

This longitudinal study adopted a quasi‐experimental design with randomized‐cluster sampling (Ager, Ager, Stavrou, & Boothby, 2011; Morris & Nguyen, 2008). In discussion with the respective village leaders, *nyumba kumi* were mapped as natural clusters within each of the five villages. Clusters were then randomly selected from each village, with the number selected proportionate to population size of the village. At baseline, all households in the randomly selected clusters were visited to identify those where children between the ages of 6 and 12 years were residing. Multiple visits to households were made as necessary to secure engagement of the primary caregiver and confirmation that the household met this inclusion criterion. If the primary caregiver consented to participation, they were invited to respond with respect to all children between the above ages residing in the household. Baseline interviews were completed in the early weeks of settlement (October–November 2012) before the opening of CFS locations. For endline assessment (which fell 3–6 months later given the phased introduction of individual CFS), these same caregivers were then interviewed during the final weeks of the CFS program in their respective villages. Caregiver reports of CFS attendance in the intervening period were collected (and triangulated with CFS attendance records where these were fully available). Caregivers were interviewed again 18 months following baseline assessment, when all formal CFS programming had ceased.

At baseline, a team of six enumerators, three from the refugee community and three from the host community, were selected based on prior experience working with children and survey data collection as well as fluency in the local dialects of *Kiswahili* and *Kinyabwisha*, both widely spoken in the settlement. Enumerators were selected and trained over the period of a week in survey protocols, mobile phone data administration, informed consent, and interview techniques. Refresher trainings were provided throughout the study period, and daily debriefings were conducted to share successes and failures of the day's efforts. This team remained throughout the study duration with the addition of two enumerators at follow‐up.

#### Consent and ethical approval

Informed consent was sought from all interviewees. Participants were asked to make their ‘mark’ or provide their signature acknowledging willingness to participate. Written copies of the consent form were available in local dialects. The study protocol was reviewed and approved by the Columbia University Medical Center IRB (Reference IRB‐AAAJ4352) and the Office of the Prime Minister of Uganda.

### Measures

Measurement tools were selected in collaboration with the program team to assess the main objectives of CFS intervention: (a) child protection, (b) supporting caregivers and communities in strengthening systems of support, care, and protection of children, and (c) the promotion of children's social and emotional well‐being. Measures were translated into the two local dialects by a trained translator and then – following piloting in one village – confirmed through group translation and back translation to ensure team comprehension and accuracy in the contextualization of core constructs.

#### Protection of children and systems of support, care, and protection

The Child Protection Rapid Assessment (CPRA) is an interagency designed tool that provides a series of questions to rapidly identify and prioritize protection needs of children in the context of a rapid‐onset emergency (Global Protection Cluster Child Protection Working Group, 2011). Items – all scored and analyzed independently – addressed caregivers protection concerns for their children and stresses related to caregiving (e.g. ‘Are there any reasons you fear for your child's safety? If so, what are they?’). The CPRA also provided questions to assess knowledge of resource persons for children's well‐being, and awareness of reporting structures and support services for children experiencing abuse or neglect (e.g. ‘If you suspected that a child in the community was being abused, where could you go to report this?’).

#### Mental health and PSWB

Two scales were used to assess the social and emotional well‐being of children. A locally derived measure of PSWB, based upon indicators suggested by extensive ethnographic fieldwork in Uganda (CPC, 2011), assessed aspects of social and emotional well‐being of children, including engagement at home, at school, and in the community; social relations; problem‐solving skills and behaviors; self‐esteem; and troubling thoughts and feelings (in questions of the form ‘Do they have many good friends?’). Higher scores are indicative of more adaptive capacities on a scale from 0 to 21.

Derived from the SEARCH Institute's 58‐item Developmental Assets Profile (2005; Scales et al., 2015), the 10‐item Caregiver Rating of Development Assets (CRDA) scale was developed for this study to explore ratings by caregivers of children's development assets, both internal (across the domains of Positive Values, Social Competencies, Positive Identity, Commitment to Learning) and external (across the domains of Support, Empowerment, Constructive Use of Time, Boundaries & Expectations). Items were of the form ‘How sensitive to the needs and feelings of others are they?’

#### Vulnerability assessment

Consistent with implementing agency policies, children were screened with respect to the following criteria deemed to be reliably assessed in the context: primary caregiver aged 65 or above, member of female‐headed household, family with over five members residing more than four nights per week in the home, physical disability of the child, and perceived functional impairment related to mental disability. Children with three or more vulnerabilities were designated as ‘vulnerable’ for the subsequent analysis.

### Data analysis

Following data collection and cleaning, to account for potential contamination, we restricted analyses to data pertaining to children residing in the same geographic areas throughout the study period. The internal consistency of measurement scales – the PSWB and CDRA – was examined using Cronbach's alpha. The intervention group was comprised of CFS attenders, defined as children reported as attending the CFS at T2 either ‘sometimes’ or ‘always’. To estimate short‐ and longer‐term impact, we compared mean scores of outcomes for CFS attenders and nonattenders at baseline and endline and at baseline and follow‐up, respectively. We began with univariate analyses (*t*‐ and chi‐square tests) and then conducted bivariate and multivariate linear regressions controlling for baseline outcome scores, age, gender, and school attendance. We calculated Cohen's d to estimate the effect of attending CFS at T2. We additionally used logistic regressions to explore what factors (age, gender, village, vulnerability status) predicted CFS attendance at T2. We further explored the effects of attending a higher‐ vs. lower‐quality CFSs in comparison with nonattendance via multivariate regressions (again controlling for baseline outcome scores, age, gender, and school attendance). Multilevel multivariate modeling, using households and village clusters as levels, was also attempted; however, sample size compromised the integrity of such models (models not reported).

## Results

### Recruitment and retention

**Figure 1:**
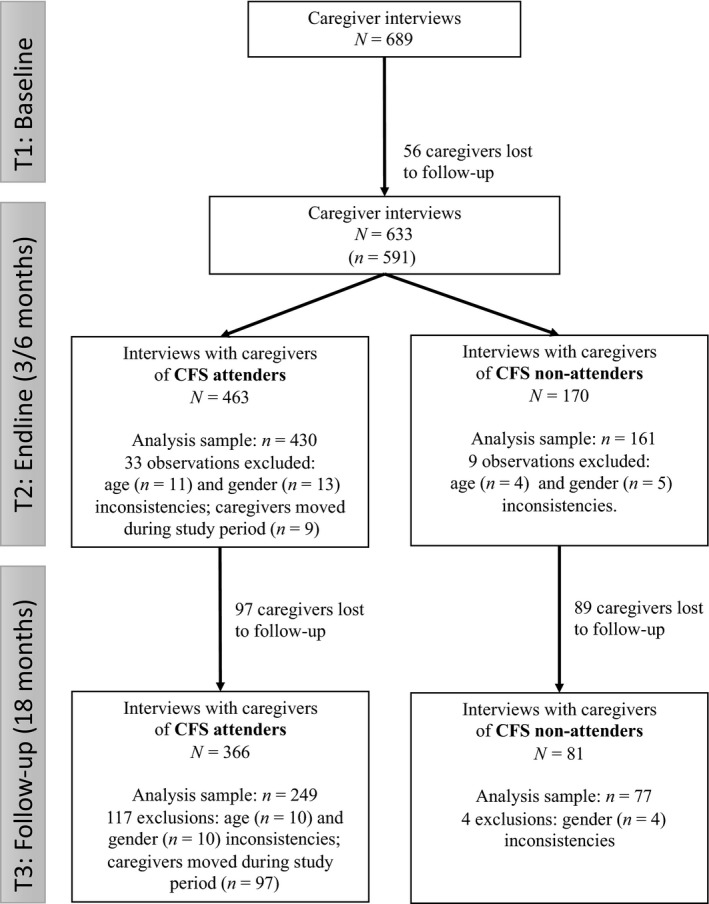
Participant recruitment and retention by study period. *N* = interviews conducted; *n* = observations included in analysis

Figure 1 summarizes recruitment and retention through the three phases of the study. At baseline (T1), caregivers reported on 689 children, of which 633 were reported on again at endline. Of these, 42 records were inconsistent in terms of a child's age or gender or, for children reported as attending CFS, reported changed location since T1 (rendering association with a specific CFS uncertain); these observations were excluded from further analyses. There were no differences at T1 between those lost to or excluded from analysis and those retained, other than the latter reporting higher levels of child protection concerns and greater knowledge of child protection resources. Of the 591 children comprising the endline cohort, 430 were CFS attenders and 161 CFS nonattenders.

At follow‐up (T3), data were collected with respect to 447 children of the original sample. We retained and analyzed data for 326 children for whom we could match records regarding age and gender across all measurement points and, for children reported as previously attending CFS, with unchanged location (enabling association with a specific CFS). 249 of these 326 (76.4%) children had previously attended CFS during their operation; 77 (23.6%) were nonattenders (see Figure 1). Baseline characteristics for the sample retained to T3 were broadly similar to those retained for T2 (see Table S1).

### Validity of measures

Internal consistency of all scales was deemed adequate for valid analysis. At baseline, Cronbach *α*‐coefficients were a moderate‐to‐strong .78 for the CRDA and an acceptable .66 for PSWB. At endline, coefficients of .77 and .74 were secured for the CRDA and PSWB, respectively, with scores of .89 for the CRDA and .91 for PSWB observed at follow‐up.

T2 caregiver reports of attendance were validated with respect to a random sample of 100 children listed on attendance registers at two CFSs where records were kept consistently. These registers indicated an average attendance of over 62% of available sessions for children reported by caregivers as attenders and less than 12% of available sessions for those reported as nonattenders.

### Short‐term analysis

#### Comparability of intervention and comparison groups

Baseline and endline characteristics for the sample retained at T2 are summarized in Table 1. At baseline, there were no statistically significant differences between those who subsequently attended CFS (intervention) or did not attend CFS (comparison) in relation to gender (χ^2^_(2, *n* = 591)_ = 3.231, *p* = .072) or village of residence (χ^2^_(4, *n* = 591)_ = 7.205, *p* = .125). Vulnerability was not a predictor of CFS attendance (OR 1.39, *p* = .196). Both children who had attended a CFS and their caregivers tended to be slightly younger than children and caregivers of those who did not attend. Children whose caregivers reported them attending school in the period between baseline and endline were less likely to have attended a CFS (OR = 0.283, *p* < .001). Scoring on protection and well‐being measures at baseline were generally similar for intervention and comparison groups. However, reported developmental assets and knowledge of child protection resources were significantly lower – and reported barriers to accessing and using these child protection resources significantly higher – for those whose children subsequently attended CFS. On the basis of these differences – and the nonrandom allocation of attenders and nonattenders – we used multivariate regressions controlling for baseline outcome scores, age, gender, and school attendance as the basis for subsequent reported findings.

**Table 1:** Short‐term analysis: sample characteristics

Characteristics of attenders and nonattenders at baseline and (T1) and endline (T2)												
Variables of interest	Baseline						Endline					
	Overall	Attenders		Nonattenders		*p*	Overall	Attenders		Nonattenders		*p*
		*N*	%	*N*	%			*N*	%	*N*	%	
Sample characteristics												
Child gender												
Female	300	228	38.58	72	12.18	.072	300					
Male	291	202	34.18	89	15.06		291					
Child age, mean (*SD*)	8.414 (1.990)	8.051 (1.862)		9.385 (1.997)		<.001	8.431 (1.995)	8.067 (1.865)		9.404 (2.011)		<.001
Biological parent status (588 at endline)												
Both living	472	343	58.04	129	21.83	.863	461	342	58.16	119	20.24	.258
One living	107	79	13.37	28	4.74		95	63	10.71	32	5.44	
Neither living	12	8	1.35	4	0.68		32	22	3.74	10	1.7	
Primary caregiver age, mean (*SD*)	36.673 (10.145)	35.721 (9.851)		39.217 (10.503)		<.001	36.617 (9.919)	35.670 (9.616)		39.149 (10.296)		<.001
Primary caregiver of child												
Mother	298	214	36.21	84	14.21	.643	306	235	39.76	71	12.01	.119
Father	264	197	33.33	67	11.34		243	169	28.6	74	12.52	
Siblings and/or aunt/uncle	15	10	66.67	5	33.33		21	14	66.67	7	33.33	
Not relative/Other	13	8	1.35	5	0.85		21	12	2.03	9	1.52	
Child vulnerability												
Vulnerable	108	84	14.21	24	4.06							
Not Vulnerable	483	346	58.54	137	23.18							

Key outcomes^a^	Overall	Attenders		Nonattenders		*p*	Overall	Attenders		Nonattenders		*p*
	Mean (*SD*)	Mean (*SD*)	*N*	Mean (*SD*)	*N*		Mean (*SD*)	Mean (*SD*)	*N*	Mean (*SD*)	*N*	
Stress												
Child protection concerns	5.527 (2.022)	5.523 (0.099)	430	5.54 (1.924)	161	.927	4.831 (1.992)	4.856 (2.026)	430	4.764 (1.906)	161	.618
Caregiver stresses	4.479 (0.823)	4.507 (0.824)	430	4.404 (0.817)	161	.175	4.002 (1.288)	4.028 (1.264)	430	3.932 (1.352)	161	.419
Mental Health and Well‐being												
Psychosocial well‐being	13.007 (3.322)	12.897 (0.157)	398	13.313 (0.314)	144	.199	12.309 (3.620)	12.651 (3.129)	424	11.41 (4.564)	161	<.001
Development												
Developmental assets	14.456 (5.368)	14.093 (5.299)	333	15.385 (5.451)	130	.020	15.089 (4.870)	15.393 (4.365)	417	14.276 (5.954)	156	.014
Child protection resources												
Knowledge of CP resources	0.514 (0.996)	0.459 (0.916)	381	0.655 (1.171)	148	.042	1.176 (1.461)	1.115 (1.432)	391	1.336 (1.527)	149	.117
Perceived barriers to accessing resources	2.850 (1.747)	2.963 (1.78)	427	2.553 (1.624)	161	.011	2.829 (1.640)	2.912 (1.594)	430	2.609 (1.743)	161	.046
Perceived barriers to known reporting mechanisms	3.337 (1.442)	3.44 (1.408)	266	3.023 (1.509)	87	.019	2.976 (1.500)	2.895 (1.458)	209	3.183 (1.512)	82	.141

a Sample sizes (*N*) refer to complete case analysis.

#### Intervention impact

**Figure 2:**
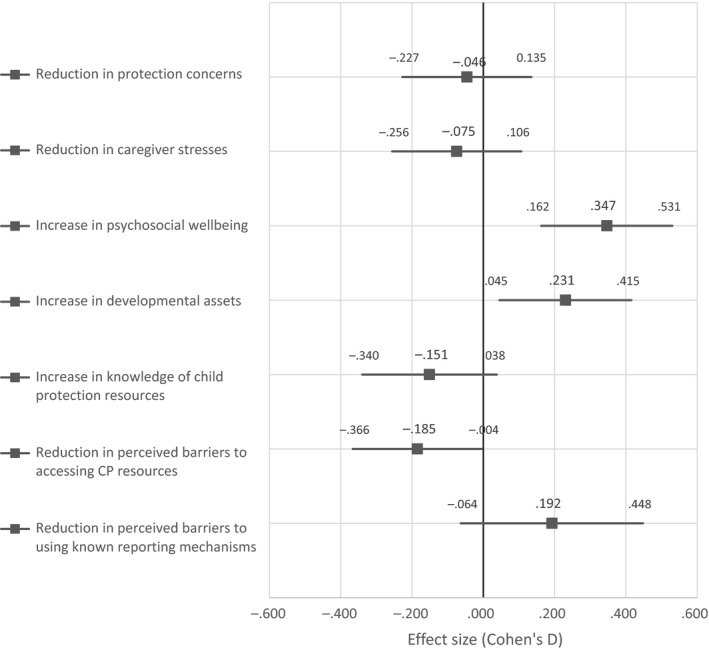
Effect size estimates of attending Child Friendly Spaces at T2 on targeted outcomes

**Figure 3:**
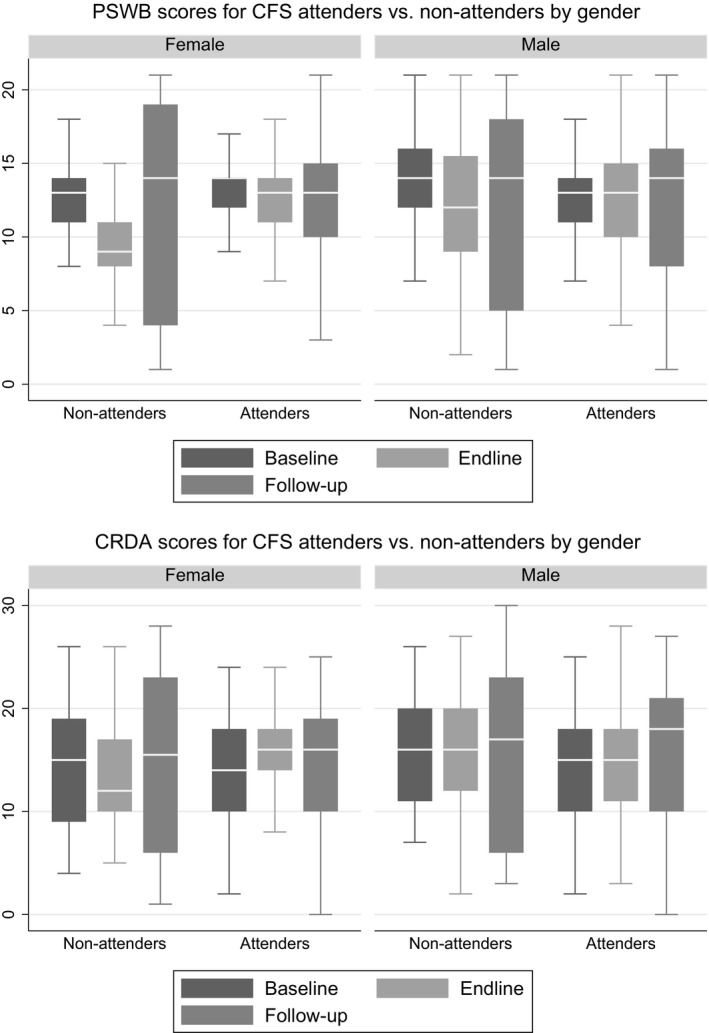
Distribution of psychosocial well‐being (PSWB) and Caregiver Rating of Development Assets (CRDA) scores at T1, T2, and T3 by attendance status

Figure 2 shows the Cohen's *D* effect‐size estimates of attending CFS on key outcomes at T2. Table 2 reports average effects of CFS attendance on key targeted outcomes at T2 obtained by multivariate analysis, controlling for variation in intervention and comparison groups on demographic variables (age and gender), baseline outcome scores, and school attendance. CFS attendance was associated with statistically significant increases in caregiver‐reported developmental assets (β = 2.517, *p* < .001, Cohen's *d* = .231) and better maintenance of PSWB (β = 2.093, *p* < .001, Cohen's *d* = .347). The latter was notably in the context of generally deteriorating well‐being scores for those not attending CFS (see Figure 3). On both measures, statistically significant impacts of CFS attendance were strongly driven by positive effects for girls (β = 3.413, *p* < .001 and β = 2.956, *p* < .001 for developmental assets and PSWB, respectively).

**Table 2:** Multivariate regressions (adjusted for baseline outcome score, age, gender, school attendance) indicating impact of Child Friendly Spaces (CFS) attendance at T2 on key outcomes at endline

Outcomes in relation to CFS attendance at T2	Beta	95‐low	95‐high	*p*	*n*
Entire cohort					
Stress					
Child protection concerns	−0.001	−0.416	0.414	.996	591
Caregiver stresses	−0.207	−0.461	0.047	.110	591
Mental Health and Well‐being					
Psychosocial well‐being^a^	2.093	1.303	2.883	<.001	536
Development					
Developmental assets^a^	2.517	1.384	3.650	<.001	449
Child protection resources					
Knowledge of CP resources	0.016	−0.308	0.340	.924	483
Perceived barriers to accessing resources	0.379	0.039	0.718	.029	588
Perceived barriers to known reporting mechanisms	−0.268	−0.885	0.350	.394	188
Girls only					
Stress					
Child protection concerns	−0.109	−0.697	0.478	.715	300
Caregiver stresses	−0.137	−0.534	0.260	.498	300
Mental Health and Well‐being					
Psychosocial well‐being^a^	2.956	1.904	4.007	<.001	275
Development					
Developmental assets^a^	3.413	1.803	5.023	<.001	222
Child protection resources					
Knowledge of CP resources	0.043	−0.442	0.529	.860	250
Perceived barriers to accessing resources	0.112	−0.375	0.599	.651	299
Perceived barriers to known reporting mechanisms	−0.629	−1.477	0.219	.144	94
Boys only					
Stress					
Child protection concerns	0.102	−0.492	0.696	.735	291
Caregiver stresses	0.122	−0.244	0.487	.513	291
Mental Health and Well‐being					
Psychosocial well‐being	1.338	0.154	2.523	.027	261
Development					
Developmental assets	1.757	0.151	3.364	.032	227
Child protection resources					
Knowledge of CP resources	−0.041	−0.479	0.396	.853	233
Perceived barriers to accessing resources	0.590	0.112	1.067	.016	289
Perceived barriers to known reporting mechanisms	−0.014	−0.904	0.876	.975	94

a Denotes outcomes where CFS attendance is statistically significant once Bonferroni correction applied; complete case analyses reported only.

The attribution of these trends to CFS attendance is supported by subgroup analysis (see Table S2), which indicated greater positive impact for those attending higher‐ versus lower‐quality CFS (all comparisons referenced to nonattenders): for PSWB, attenders of high‐quality CFS (β = 2.603, *p* < .001) vs. attenders of lower‐quality CFS (β = 1.793, *p* < .001); for CRDA, attenders of higher‐quality CFS (β = 2.942, *p* < .001) vs. attenders of lower‐quality CFS (β = 2.337, *p* < .001). Indeed, amongst boys, benefits of program attendance were only indicated for those attending higher‐quality CFS (β = 2.084, *p* = .006 for PSWB).

Overall, there was reduced reporting of protection concerns and caregiver stress between baseline and endline, and caregiver knowledge of child protection resource persons more than doubled (see Table 1). Figure 2 provides little evidence of differential impact for caregivers of CFS attending and nonattending children on these measures; multivariate analysis confirmed no statistically significant effects in relation to these variables (see Table 2).

### Longer‐term analysis

#### Intervention impact

At follow‐up, no differential outcomes by CFS status were observed. Table 3 summarizes variation in targeted outcomes at T3 by CFS attendance status. Endline differences between attenders and nonattenders were nonsignificant for PSWB (β = 1.324 *p* = .985) and development assets (β = 2.109, *p* = .074), with no significant impact of prior CFS attendance identified for girls (for whom, effects have been strongest at T2) or attenders of higher‐quality CFS (see Table 3 and Table S3, respectively). This lack of longer‐term intervention impact was in a context of a general increase in reported protection concerns (a 14% increase from T1 to T3) and a fourfold increase from baseline to follow‐up of caregiver knowledge of resources available to support children.

**Table 3:** Multivariate regressions (adjusted for baseline outcome score, age, gender, school attendance) indicating impact of Child Friendly Spaces (CFS) attendance at T2 on key outcomes at follow‐up^a^

Key outcomes	Follow‐up				
	Beta	95‐low	95‐high	*p*	*n*
Entire cohort					
Stress					
Child protection concerns	−0.394	−1.413	0.624	.447	326
Caregiver stresses	0.004	−0.402	0.410	.985	326
Mental Health and Well‐being					
Psychosocial well‐being	1.324	−0.391	3.038	.130	306
Development					
Developmental assets	2.109	−0.208	4.426	.074	251
Child protection resources					
Knowledge of CP resources	0.123	−0.491	0.737	.694	281
Perceived barriers to accessing resources	0.098	−0.462	0.659	.730	324
Perceived barriers to known reporting mechanisms	0.062	−0.703	0.827	.873	148
Girls only					
Stress					
Child protection concerns	−0.287	−1.774	1.200	.704	164
Caregiver stresses	0.023	−0.557	0.602	.938	164
Mental Health and Well‐being					
Psychosocial well‐being	1.811	−0.642	4.264	.147	151
Development					
Developmental assets	2.667	−0.529	5.863	.101	122
Child protection resources					
Knowledge of resource persons	−0.247	−1.165	0.671	.596	144
Perceived barriers to accessing resources	0.058	−0.735	0.850	.886	164
Perceived barrier to known reporting mechansisms	0.487	−0.621	1.595	.383	68
Boys only					
Stress					
Child protection concerns	−0.545	−1.959	0.869	.448	162
Caregiver stresses	−0.038	−0.622	0.547	.899	162
Mental Health and Well‐being					
Psychosocial well‐being	0.843	−1.664	3.351	.507	155
Development					
Developmental assets	1.525	−1.902	4.951	.380	129
Child protection resources					
Knowledge of CP resources	0.493	−0.338	1.324	.243	137
Perceived barriers to accessing resources	0.149	−0.654	0.951	.715	160
Perceived barriers to known reporting mechanisms	−0.307	−1.397	0.783	.576	80

a Denotes outcomes where CFS attendance is statistically significant once Bonferroni correction applied; complete case analyses reported only.

Figure 3 shows the distribution of PSWB and development asset scores over time for CFS attenders and nonattenders. As indicated earlier, despite lower overall scores at baseline, CFS attenders were reported to have better PSWB and greater development assets than nonattenders at endline of the intervention period. At follow‐up, however, scores on both variables had returned close to baseline levels. The substantial increase in variance of scores at follow‐up – especially amongst nonattenders – suggests an increasing diversity in the trajectories of children as they entered the second year of their displacement from DRC Congo.

Regression analyses confirmed that neither baseline characteristics of gender and vulnerability, nor baseline scores on well‐being and development assets, predicted scores on these outcomes at T3. Age was found to be the sole significant predictor of a T3 outcome, with respect to development assets (β* *= 0.658, *p* = .009).

## Discussion

In the early months of establishing the Rwamwanja Refugee Settlement, the studied CFSs provided structured activities for some three‐quarters of those within the target age range in their catchment area. Our findings suggest that this CFS programming contributed to protecting PSWB and development assets for these children in this period. This is fully in line with the humanitarian imperative to protect, relieve suffering and respect dignity in crisis settings (ICRC, 1994). However, it is clear from our findings that these intervention impacts are not sustained. By follow‐up, previous attenders and nonattenders were essentially indistinguishable on these measures. This lack of durability of intervention impacts to follow‐up cautions against the presentation of CFS as a programming approach influencing longer‐term developmental trajectories of displaced children.

Two elaborations of this conclusion need to be made, however, for appropriate interpretation. First, the loss of intervention impact was not as a result of loss of gains by CFS attenders, but by improvements for nonattenders over the longer‐term. Other mechanisms of adaptation – drawing on familial and community resources – were apparently driving gains for all children, a common observation in studies rigorously examining progress of comparison groups (Ager & Metzler, 2017). Second, the greater variance observed on these measures at follow‐up – particularly amongst nonattenders – suggests increasingly divergent trajectories of adaptation by follow‐up. While some nonattenders had adapted well to life in the settlement area, a significant minority were reporting poor well‐being and major lack of development assets. This ‘tail’ of the distribution, indicating the emergence of a group of children and caregivers really struggling with life in the settlement area after the majority of emergency reception programs have closed, represents a major humanitarian concern.

The lack of short‐term impact of CFS attendance on protection concerns and caregiver stresses needs to be interpreted in the context of the wider risks presenting within the settlement (such as forced recruitment, attacks, abductions, sexual violence), which it is challenging for sessional programming to address. Significant protection impacts of CFS have, however, been reported in other contexts (e.g. Metzler et al., 2013, for Somali refugees in Ethiopia, where fenced compounds provided a strong sense of security in the face of potential border incursions by *Al‐Shabaab*).

The overall trends over the course of the study for increased knowledge of protection resources, and reductions in perceived barriers to resource access and reporting, amongst caregivers suggest growing mobilization of resources to support and protect children at Rwamwanja. The place of CFS in this process of mobilization is, however, unclear. There were a number of other agencies operating in Rwamwanja – during the CFS intervention period in particular – whose work could have contributed to gains in this area. Further, there is clearly greater opportunity for knowledge about resources to diffuse from CFS attenders to nonattenders than is the case for other targeted outcomes, rendering a further challenge to attributing change. An alternative approach to research design would be required to more confidently discern influences on processes of mobilization of resources in such circumstances.

In terms of implications for implementation of programming to support the protection and well‐being of children displaced through humanitarian crisis, we have previously highlighted the need to address the subgroup of children with deteriorating trajectories of adjustment over time through targeted provision continuing beyond an initial settlement period. It is also clear that if CFS interventions are to have impact beyond their period of delivery, they need to be more purposeful in securing effective linkage with resources available to sustain such influence. In the context of Rwamwanja – as in many refugee settings – schools would have provided the most tangible basis for this engagement. Indeed, our findings suggest that those that had secured a place at the one school serving the settlement area were four‐times less likely to attend CFS. More broadly, if CFSs are to provide a basis for outreach, partnership with communities (including parents) toward the support and protection of children must remain central to the design and implementation of the intervention. It is vital to provide integrated services that remain flexible to the evolving needs and priorities of children and families and are not dependent on unsustainable external programming.

Finally, the observation of stronger intervention effects on PSWB and development assets for CFS programming that met high programing standards underlines the general importance of ensuring quality and fidelity in interventions implemented at scale if targeted impacts are to be secured. Planned work examining the impacts of alternative approaches to CFS curricula builds upon this finding (Savage & Metzler, 2018).

This study has clear limitations. First, and most importantly, delays in program implementation and the mobility of children across the settlement prevented the randomization of sample areas to intervention and waitlist control as initially planned. Counterfactual analysis thus had to be conducted through the alternative means described: multivariate analysis controlling for variation between the groups on demographic characteristics and baseline outcomes. The observation of greater positive impact on well‐being and development assets for those attending higher‐ versus lower‐quality CFS clearly supports the attribution of change on these variables to aspects of CFS programming. Second, the lack of consistent record‐keeping by most CFSs resulted in reliance on caregiver self‐report of CFS attendance. Although triangulation with CFS attendance records at two CFSs that had maintained regular record‐keeping suggested that self‐report was a valid basis to identify attenders vs. nonattenders, more fine‐grained analysis of influence of rates of attendance was not possible. Third, with the lack of a comprehensive listing of households to support random sampling, equiprobability of selection of households was reliant on a cluster sampling approach designed for humanitarian settings (Morris & Nguyen, 2008). Fourth, with no feasible means in this humanitarian setting of preventing diffusion of knowledge secured through CFS attendance to nonattenders, the potential influence of CFS programming on resource mobilization regarding child protection in the settlement is potentially underestimated.

Notwithstanding these limitations, the present study represents the most rigorous attempt to date to measure the short‐ and longer‐term impacts of implementing CFS in a humanitarian context. Findings suggest a clear short‐term impact on PSWB and development assets that fulfills the humanitarian mandate to relieve suffering in such contexts. Claims for longer‐term impacts on children's trajectories were, in this instance, largely unsubstantiated. Strengthening strategies of community engagement appear key to achieving longer‐term impacts in addition to important short‐term ones.

**Key points**

- In response to the protection and psychosocial support needs of refugee children, humanitarian agencies regularly establish activities in child friendly spaces (CFSs). However, there is little evidence of their impact in either the short‐ or longer‐term.
- This study reports on caregivers reports of children’s wellbeing – and their concerns for their children ‐ on first arrival in the studied refugee settlement (before the commencement of CFS programming); at the end of the intervention period; and at one‐year follow‐up.
- Those children that attended a CFS maintained better psychosocial wellbeing and secured greater development assets after 3 to 6 months, with the strongest impacts in the better‐run CFSs.
- A further year later, however, there were no significant differences between children who had attended or not attended a CFS in their early months in the refugee settlement, although scoring in the latter showed greater variation.
- If interventions in humanitarian contexts are to substantially influence longer‐term trajectories of well‐being and adjustment they will need to be more effective in connecting children to resources in conditions of protracted displacement.

## Supporting information


**Table S1.** Longer‐term analysis: sample characteristics.
**Table S2.** Multivariate regressions (adjusted for baseline outcome score, age, gender, school attendance) indicating impact of lower and higher‐quality CFS attendance at T2 on key outcomes at endline.
**Table S3.** Multivariate regressions (adjusted for baseline outcome score, age, gender, school attendance) indicating impact of lower and higher‐quality CFS attendance at T2 on key outcomes at follow‐up.

## References

[jcpp13069-bib-0001] Ager, A. , Ager, W. , Stavrou, V. , & Boothby, N. (2011). Inter‐agency guide to the evaluation of psychosocial programming in emergencies. New York: UNICEF.

[jcpp13069-bib-0002] Ager, A. , Blake, C. , Stark, L. , & Daniel, T. (2011). Child protection assessment in humanitarian emergencies: Case studies from Georgia, Gaza, Haiti and Yemen. Child Abuse and Neglect, 35, 1045–1052. 22099145 10.1016/j.chiabu.2011.08.004

[jcpp13069-bib-0003] Ager, A. , & Metzler, J. (2017). Where there is no intervention: Insights into processes of resilience supporting war‐affected children. Peace and Conflict: Journal of Peace Psychology, 23, 67–75.

[jcpp13069-bib-0004] Ager, A. , Metzler, J. , Vojta, M. , & Savage, K. (2013). Child friendly spaces: A systematic review of the current evidence‐base on outcomes and impact. Intervention, 11, 133–148.

[jcpp13069-bib-0005] Bangpan, M. , Dickson, K. , Felix, L. , & Chiumento, A. (2017). The impact of mental health and psychosocial support interventions on people affected by humanitarian emergencies: A systematic review humanitarian evidence programme. Oxford, UK: Oxfam GB.

[jcpp13069-bib-0006] Betancourt, T. , Meyers‐Ohki, S. , Charrow, A. , & Tol, W.A. (2013). Interventions for children affected by war: An ecological perspective on psychosocial support and mental health care. Harvard Review of Psychiatry, 21, 70–91. 23656831 10.1097/HRP.0b013e318283bf8f PMC4098699

[jcpp13069-bib-0007] Betancourt, T. , Salhi, C. , Buka, S. , Leaning, J. , Dunn, J. , & Earls, F. (2011). Connectedness, social support and internalising emotional and behavioural problems in adolescents displaced by the Chechen conflict. Disasters, 36, 635–655. 10.1111/j.1467-7717.2012.01280.x PMC3735440 22443099

[jcpp13069-bib-0008] Blanchet, K. , Ramesh, A. , Frison, S. , Warren, E. , Hossain, M. , Smith, J. , … & Roberts, B. (2017). Evidence on public health interventions in humanitarian crises. The Lancet, 390, 2287–2296. 10.1016/S0140-6736(16)30768-1 28602563

[jcpp13069-bib-0009] Brown, F. , de Graaff, A. , Annan, J. , & Betancourt, T. (2016). Annual Research Review: Breaking cycles of violence – A systematic review and common practice elements analysis of psychosocial interventions for children and youth affected by armed conflict. Journal of Child Psychology and Psychiatry, 58, 507-524. 27943284 10.1111/jcpp.12671

[jcpp13069-bib-0010] Child Protection in Crisis (CPC) Learning Network . (2011). Defining success: Developing locally meaningful indicators for child‐centred psychosocial programming in Uganda. New York: Columbia University, Child Protection in Crisis Learning Network.

[jcpp13069-bib-0011] Global Protection Cluster Child Protection Working Group (2011). Child protection rapid assessment toolkit. Geneva, Switzerland: CPWG.

[jcpp13069-bib-0012] Global Protection Cluster Child Protection Working Group (2015). A matter of life and death: Child protection programming’s essential role in ensuring child wellbeing and survival during and after emergencies. Geneva, Switzerland: CPWG.

[jcpp13069-bib-0013] Global Education Cluster, Global Protection Cluster, INEE and UASC (2011). Guidelines for child friendly spaces in emergencies. New York, NY: Global Education Cluster.

[jcpp13069-bib-0014] International Committee of the Red Cross & ICRC (1994). The code of conduct for international red cross and red crescent movement and NGOs in disaster relief. Geneva, Switzerland: International Committee of the Red Cross and ICRC.

[jcpp13069-bib-0015] IRIN . (2012). North Kivu in turmoil again. Available from: http://www.irinnews.org/report/95465/drc-north-kivu-turmoil-again [last accessed 27 August 2016].

[jcpp13069-bib-0016] International Union for Conservation of Nature, United Nations Environment Programme & Rwenzo-Green Associates. (2013). Environmental and Social Impact Statement for the Rwamwanja Refugee Settlement, Kamwenge District. Available from: http://www.adansonia-consulting.ch/document/FINAL%20Report%20ESIA%20FOR%20RS%2026-2-2013.pdf [last accessed 27 April 2019].

[jcpp13069-bib-0017] Jordans, M.J.D. , Tol, W.A. , Komproe, I.H. , & de Jong, J.T.V.M. (2009). Systematic review of evidence and treatment approaches: Psychosocial and mental health care for children in war. Child and Adolescent Mental Health, 14, 2–14.

[jcpp13069-bib-0018] Kostelny, K. , & Wessells, M. (2008). The protection and psychosocial wellbeing of young children following armed conflict: Outcome research on Child‐Centered Spaces in Northern Uganda. Journal of Developmental Processes, 3, 13–25.

[jcpp13069-bib-0019] Kostelny, K. , & Wessells, M. (2013). Child friendly spaces: Promoting children's resiliency amidst war In Fernando C. & Ferrari M. (Eds.), Handbook of resilience in children of war (pp. 119–129). New York: Springer.

[jcpp13069-bib-0020] Madfis, J. , Martyris, D. , & Triplehorn, C. (2010). Emergency safe spaces in Haiti and the Solomon Islands. Disasters, 34, 845–864. 20345461 10.1111/j.1467-7717.2010.01172.x

[jcpp13069-bib-0021] Masten, A.S. , & Narayan, A. (2012). Child development in the context of disaster, war, and terrorism: Pathways of risk and resilience. Annual Review of Psychology, 63, 227–257. 10.1146/annurev-psych-120710-100356 PMC5858878 21943168

[jcpp13069-bib-0022] Metzler, J. , Savage, K. , Vojta, M. , Yamano, M. , Schafer, A. , & Ager, A. (2013). Evaluation of child friendly spaces: Ethiopia field study summary report. World Vision International & Columbia University Mailman School of Public Health.

[jcpp13069-bib-0023] Miller, K.E. , & Rasmussen, A. (2010). War exposure, daily stressors, and mental health in conflict and post‐conflict settings: Bridging the divide between trauma‐focuses and psychosocial frameworks. Social Science and Medicine, 70, 7–16. 19854552 10.1016/j.socscimed.2009.09.029

[jcpp13069-bib-0024] Morris, S.K. , & Nguyen, C.K. (2008). A review of the cluster survey sampling method in humanitarian emergencies. Public Health Nursing, 25, 370–374. 18666943 10.1111/j.1525-1446.2008.00719.x

[jcpp13069-bib-0025] Reed, R. , Fazel, M. , Jones, L. , Panter‐Brick, C. , & Stein, A. (2011). Mental health of displaced and refugee children resettled in low and middle‐income countries: Risk and protective factors. The Lancet, 379, 250–265. 10.1016/S0140-6736(11)60050-0 21835460

[jcpp13069-bib-0026] Savage, K. , & Metzler, J. (2018). An RCT of enhanced Child Friendly Space interventions for children affected by conflict and displacement. Cardiff, UK: Elrha Available from: http://www.elrha.org/map-location/randomised-control-trial-of-enhanced-child-friendly-space-interventions-for-girls-and-boys-affected-by-conflict-and-displacement/ [last accessed 27 April 2019].

[jcpp13069-bib-0027] Save the Children . (2008). Child friendly spaces in emergencies: A handbook for save the children staff. Available from: https://resourcecentre.savethechildren.net/library/child-friendly-spaces-emergencieshandbook-save-children-staff [last accessed 27 April 2019].

[jcpp13069-bib-0028] Save the Children . (2009). Child friendly spaces facilitator training manual. Available from: https://resourcecentre.savethechildren.net/library/child-friendly-spaces-facilitator-training-manual [last accessed 15 April 2019].

[jcpp13069-bib-0029] Save the Children Sweden . (2010). Child friendly spaces handbook for animators (Volunteers). North Darfur program. Stockholm, Sweden: Save the Children Sweden.

[jcpp13069-bib-0030] Scales, P.C. , Roehlkepartain, E.C. , Wallace, T. , Inselman, A. , Stephenson, P. , & Rodriguez, M. (2015). Brief report: Assessing youth wellbeing in global emergency settings: Early results from the emergency developmental assets profile. Journal of Adolescence, 45, 98–102. 26426457 10.1016/j.adolescence.2015.09.002

[jcpp13069-bib-0031] Search Institute . (2005). Developmental assets profile. User's manual. Minneapolis, MN: Search Institute.

[jcpp13069-bib-0032] Tol, W.A. , Barbui, C. , Galappatti, A. , Silove, D. , Betancourt, T.S. , Souza, R. , … & van Ommeren, M. (2011). Mental health and psychosocial support in humanitarian settings: Linking practice and research. The Lancet, 378, 1581–1591. 10.1016/S0140-6736(11)61094-5 PMC3985411 22008428

[jcpp13069-bib-0033] Tol, W.A. , Patel, V. , Tomlinson, M. , Baingana, F. , Galappatti, A. , Panter‐Brick, C. , … & Van Ommeren, M. (2011). Research priorities for mental health and psychosocial support in humanitarian settings. PLoS Medicine, 8, e1001096. 21949644 10.1371/journal.pmed.1001096 PMC3176752

[jcpp13069-bib-0034] Tol, W.A. , Song, S. , & Jordans, M.J.D. (2013). Resilience and mental health in children and adolescents living in areas of armed conflict—A systematic review of findings in low‐ and middle‐income countries. Journal of Child Psychology and Psychiatry, 54, 445–460. 23414226 10.1111/jcpp.12053

[jcpp13069-bib-0035] UNHCR . (2012a). Emergency response appeal to the situation in the eastern democratic Republic of the Congo. Donor Relations and Resource Mobilization Service, UNHCR.

[jcpp13069-bib-0036] UNHCR . (2012b). More than 3,000 Congolese flee to Uganda to escape clashes. Available from: http://www.refworld.org/docid/50654cd52.html [last accessed 27 August 2016].

[jcpp13069-bib-0037] UNHCR . (2014). Uganda fact sheet. Available from: http://data.unhcr.org/SouthSudan/download.php?xml:id=1382 [last accessed 27 August 2016].

[jcpp13069-bib-0038] UNHCR . (2017). DRC regional refugee response information sharing portal. Available from: http://data.unhcr.org/drc/regional.php [last accessed 19 July 2017].

[jcpp13069-bib-0039] UNICEF (2009). A practical guide for developing Child Friendly Spaces. New York: UNICEF.

[jcpp13069-bib-0040] Wessells, M. , & Kostelny, K. (2013). Child friendly spaces: Toward a grounded, community‐based approach for strengthening child protection practice in humanitarian crises. Child Abuse & Neglect, 37, 29–40. 24268375 10.1016/j.chiabu.2013.10.030

[jcpp13069-bib-0041] World Vision and Columbia University (2015). Evaluation of child friendly spaces: tools and guidance for monitoring and evaluating CFS **.** Available from: https://www.wvi.org/united-nations-and-global-engagement/publication/evaluation-child-friendly-spaces-tools-and-guidance [last accessed 27 April 2019].

[jcpp13069-bib-0042] World Vision International (2006). Children in emergencies manual. Geneva, Switzerland: WVI.

