# Supplementary material for: Short‐ and longer‐term impacts of Child Friendly Space Interventions in Rwamwanja Refugee Settlement, Uganda
Source: J Child Psychol Psychiatry. 2019 May 20;60(11):1152–63. doi: 10.1111/jcpp.13069 (PMC6852245; doi:10.1111/jcpp.13069)
Supplement: Supplementary file 1 — Table S1. Longer‐term analysis: sample characteristics. Table S2. Multivariate regressions (adjusted for baseline outcome score, age, gender, school attendance) indicating impact of lower and higher‐quality CFS attendance at T2 on key outcomes at endline. Table S3. Multivariate regressions (adjusted for baseline outcome score, age, gender, school attendance) indicating impact of lower and higher‐quality CFS attendance at T2 on key outcomes at follow‐up. [file JCPP-60-1152-s001.docx]

**Supporting information – Short- and longer-term impacts of Child Friendly Space Interventions in Rwanwanja Refugee Settlement, Uganda – by Metzler et al.**

**Table S1: Longer-term analysis: sample characteristics**

| **Variables of interest** | **Baseline** | | | | | | **Endline** | | | | | | **Follow-up** | | | | | |
| --- | --- | --- | --- | --- | --- | --- | --- | --- | --- | --- | --- | --- | --- | --- | --- | --- | --- | --- |
|  | **Overall** | **Attenders** | | **Non-attenders** | |  | **Overall** | **Attenders** | | **Non-attenders** | |  | **Overall** | **Attenders** | | **Non-attenders** | | **p** |
|  |  | **N** | **%** | **N** | **%** | **p** |  | **N** | **%** | **N** | **%** | **p** |  | **N** | **%** | **N** | **%** |  |
| ***Sample characteristics*** |  |  |  |  |  |  |  |  |  |  |  |  |  |  |  |  |  |  |
| Child gender |  |  |  |  |  | 0.651 |  |  |  |  |  |  |  |  |  |  |  |  |
| Female | 164 | 127 | 38.96 | 37 | 6.26 |  |  |  |  |  |  |  |  |  |  |  |  |  |
| Male | 162 | 122 | 37.42 | 40 | 12.27 |  |  |  |  |  |  |  |  |  |  |  |  |  |
| Child age, mean (SD) | 8.334 (1.983) | 7.984 (0.118) |  | 9.467 (0.221) |  | <0.001 | 8.337 (1.987) | 7.988 (0.118) |  | 9.467 (0.223) |  | <0.001 | 9.319 (1.982) | 8.960 (0.116) |  | 10.481 (1.991) |  | <0.001 |
| Biological parent status (324 at follow-up) |  |  |  |  |  | 0.843 |  | //No change |  |  |  |  |  |  |  |  |  | 0.944 |
| both living | 273 | 209 | 64.11 | 64 | 19.63 |  | 0 |  |  |  |  |  | 263 | 202 | 62.35 | 61 | 18.83 |  |
| one living | 47 | 36 | 11.04 | 11 | 3.37 |  | 0 |  |  |  |  |  | 56 | 42 | 12.96 | 14 | 4.32 |  |
| neither living | 6 | 4 | 1.23 | 2 | 0.61 |  | 0 |  |  |  |  |  | 5 | 4 | 1.23 | 1 | 0.31 |  |
| Primary caregiver age, mean (SD) | 37.043 (9.849) | 35.721(9.851) |  | 40.584 (1.246) |  | <0.001 | 36.880 (9.667) | 36.177 (0.587) |  | 39.156 (1.208) |  | 0.018 | 36.383 (12.907) | 35.032 (0.771) |  | 40.753 (1.629) |  | <0.001 |
| Primary caregiver of child |  |  |  |  |  | 0.078 |  |  |  |  |  | 0.108 |  |  |  |  |  | 0.047 |
| Mother | 148 | 118 | 36.2 | 30 | 9.2 |  | 153 | 124 | 38.04 | 29 | 8.9 |  | 154 | 127 | 38.96 | 27 | 8.28 |  |
| Father | 165 | 124 | 38.04 | 41 | 12.58 |  | 158 | 116 | 35.58 | 42 | 12.88 |  | 158 | 112 | 34.36 | 46 | 14.11 |  |
| Siblings and/or aunt/uncle | 16 | 10 | 62.5 | 6 | 37.5 |  | 19 | 9 | 47.36842105 | 10 | 52.63157895 |  | 14 | 10 | 71.42857143 | 4 | 28.57142857 |  |
| Not relative/Other | 9 | 6 | 1.84 | 3 | 0.92 |  | 8 | 5 | 1.53 | 3 | 0.92 |  | 11 | 8 | 2.45 | 3 | 0.92 |  |
| Child vulnerability |  |  |  |  |  |  |  |  |  |  |  |  |  |  |  |  |  |  |
| Vulnerable | 52 | 45 | 13.80 | 7 | 2.15 |  |  |  |  |  |  |  |  |  |  |  |  |  |
| Not Vulnerable | 274 | 204 | 62.58 | 70 | 21.47 |  |  |  |  |  |  |  |  |  |  |  |  |  |
| **Key outcomes** | **Overall** | **Attenders** | | **Non-attenders** | |  | **Overall** | **Attenders** | | **Non-attenders** | |  | **Overall** | **Attenders** | | **Non-attenders** | |  |
|  | **Mean (SD)** | **Mean (SD)** | **N** | **Mean (SD)** | **N** | **p** | **Mean (SD)** | **Mean (SD)** | **N** | **Mean (SD)** | **N** | **p** | **Mean (SD)** | **Mean (SD)** | **N** | **Mean (SD)** | **N** | **p** |
| ***Stress*** |  |  |  |  |  |  |  |  |  |  |  |  |  |  |  |  |  |  |
| Child protection concerns | 5.524 (2.102) | 5.437 (0.132) | 249 | 5.805 (0.243) | 77 | 0.180 | 4.806 (2.043) | 4.839 (0.130) | 249 | 4.701 (0.228) | 77 | 0.605 | 6.294 (3.408) | 6.036 (0.216) | 249 | 7.13 (0.376) | 77.0000 | 0.014 |
| Caregiver stresses | 4.543 (0.790) | 4.566 (0.051) | 249 | 4.468 (0.086) | 77 | 0.338 | 3.954 (1.231) | 3.952 (0.079) | 249 | 3.961 (0.135) | 77 | 0.954 | 4 (1.361) | 3.952 (0.079) | 249 | 3.961 (0.135) | 77.0000 | 0.954 |
| ***Mental Health and Well-being*** |  |  |  |  |  |  |  |  |  |  |  |  |  |  |  |  |  |  |
| Psychosocial well-being | 12.926 (3.277) | 12.87 (0.197) | 239 | 13.110 (0.465) | 73 | 0.586 | 12.231 (3.773) | 12.621 (0.211) | 248 | 10.974 (0.542) | 77 | 0.008 | 12.035 (5.561) | 12.099 (0.326) | 243 | 11.822 (0.816) | 73.0000 | 0.710 |
| ***Development*** |  |  |  |  |  |  |  |  |  |  |  |  |  |  |  |  |  |  |
| Developmental assests | 14.367 (5.385) | 14.021 (0.385) | 187 | 15.304 (0.679) | 69 | 0.091 | 15.119 (5.034) | 15.302 (0.297) | 242 | 14.539 (0.709) | 76 | 0.250 | 15.057 (7.104) | 15.017 (0.423) | 242 | 15.187 (0.998) | 75.0000 | 0.857 |
| ***Child protection resources*** |  |  |  |  |  |  |  |  |  |  |  |  |  |  |  |  |  |  |
| Knowledge of CP resources | 0.527 (1.004) | 0.507 (1.013) | 223 | 0.592 (0.979) | 71 | 0.536 | 1.121 (1.433) | 0.965 (1.296) | 226 | 1.620 (1.719) | 71 | <0.001 | 2.731 (1.974) | 2.686 (1.909) | 239 | 2.877 (2.179) | 73.0000 | 0.471 |
| Perceived barriers to accessing resources | 2.781 (1.756) | 2.854 (0.113) | 247 | 2.545 (0.193) | 77 | 0.178 | 2.791 (1.647) | 2.859 (0.101) | 249 | 2.571 (0.204) | 77 | 0.18 | 2.386 (1.842) | 2.386 (0.116) | 249 | 2.39 (0.218) | 77.0000 | 0.987 |
| Perceived barriers to known reporting mechanisms | 3.256 (1.451) | 3.336 (0.117) | 147 | 3.023 (0.236) | 44 | 0.224 | 2.950 (1.462) | 2.937 (0.137) | 112 | 2.978 (0.224) | 45 | 0.8766 | 2.738 (1.638) | 2.774 (0.125) | 177 | 2.633 (0.206) | 60.0000 | 0.567 |

**Table S2:** Multivariate regressions (adjusted for baseline outcome score, age, gender, school attendance) indicating impact of lower and higher quality CFS attendance at T2 on key outcomes at endline

| **Key Outcomes at Endline** | **CFS designated as low quality** | | | | **CFS designated as high quality** | | | | **n** |
| --- | --- | --- | --- | --- | --- | --- | --- | --- | --- |
|  | **Beta** | **95-low** | **95-high** | **p** | **Beta** | **95-low** | **95-high** | **p** |  |
| **Entire cohort** | | | | | | | | | |
| ***Stress*** |  |  |  |  |  |  |  |  |  |
| Child protection concerns | -0.035 | -0.494 | 0.424 | 0.880 | -0.048 | -0.526 | 0.431 | 0.845 | 573 |
| Caregiver stresses | -0.106 | -0.402 | 0.190 | 0.481 | 0.037 | -0.272 | 0.345 | 0.814 | 573 |
| ***Mental Health and Well-being*** |  |  |  |  |  |  |  |  |  |
| Psychosocial well-being* | **1.793** | **0.935** | **2.651** | **<0.001** | **2.603** | **1.672** | **3.534** | **<0.001** | **521** |
| ***Development*** |  |  |  |  |  |  |  |  |  |
| Developmental assests* | **2.337** | **1.094** | **3.580** | **<0.001** | **2.942** | **1.620** | **4.265** | **<0.001** | **439** |
| ***Child protection resources*** |  |  |  |  |  |  |  |  |  |
| Knowledge of CP resources | 0.067 | -0.296 | 0.431 | 0.715 | 0.007 | -0.366 | 0.380 | 0.970 | 471 |
| Perceived barriers to accessing resources | 0.438 | 0.063 | 0.813 | 0.022 | 0.200 | -0.191 | 0.592 | 0.315 | 580 |
| Perceived barriers to known reporting mechanisms | -0.364 | -1.043 | 0.315 | 0.292 | -0.402 | -1.105 | 0.301 | 0.260 | 180 |
| **Girls only** | | | | | | | | | |
| ***Stress*** |  |  |  |  |  |  |  |  |  |
| Child protection concerns | -0.095 | -0.739 | 0.550 | 0.772 | -0.205 | -0.858 | 0.448 | 0.537 | 293 |
| Caregiver stresses | -0.355 | -0.787 | 0.077 | 0.107 | 0.019 | -0.419 | 0.457 | 0.932 | 293 |
| ***Mental Health and Well-being*** |  |  |  |  |  |  |  |  |  |
| Psychosocial well-being* | **2.799** | **1.659** | **3.939** | **<0.001** | **3.282** | **2.093** | **4.470** | **<0.001** | **269** |
| ***Development*** |  |  |  |  |  |  |  |  |  |
| Developmental assests* | **3.401** | **1.641** | **5.161** | **<0.001** | **3.481** | **1.687** | **5.276** | **<0.001** | **220** |
| ***Child protection resources*** |  |  |  |  |  |  |  |  |  |
| Knowledge of CP resources | 0.085 | -0.455 | 0.625 | 0.756 | 0.056 | -0.489 | 0.602 | 0.839 | 245 |
| Perceived barriers to accessing resources | 0.102 | -0.430 | 0.633 | 0.707 | 0.016 | -0.527 | 0.559 | 0.954 | 292 |
| Perceived barriers to known reporting mechanisms | -0.717 | -1.647 | 0.213 | 0.129 | -0.797 | -1.769 | 0.175 | 0.107 | 89 |
| **Boys only** | | | | | | | | | |
| ***Stress*** |  |  |  |  |  |  |  |  |  |
| Child protection concerns | 0.016 | -0.648 | 0.680 | 0.961 | 0.121 | -0.594 | 0.835 | 0.740 | 280 |
| Caregiver stresses | 0.114 | -0.296 | 0.524 | 0.584 | -0.011 | -0.451 | 0.429 | 0.961 | 280 |
| ***Mental Health and Well-being*** |  |  |  |  |  |  |  |  |  |
| Psychosocial well-being* | 0.914 | -0.374 | 2.203 | 0.164 | **2.084** | **0.609** | **3.558** | **0.006** | 252 |
| ***Development*** |  |  |  |  |  |  |  |  |  |
| Developmental assests | 1.413 | -0.359 | 3.185 | 0.118 | 2.481 | 0.499 | 4.462 | 0.014 | 219 |
| ***Child protection resources*** |  |  |  |  |  |  |  |  |  |
| Knowledge of CP resources | 0.018 | -0.478 | 0.513 | 0.943 | -0.063 | -0.579 | 0.453 | 0.811 | 226 |
| Perceived barriers to accessing resources | 0.723 | 0.189 | 1.257 | 0.008 | 0.339 | -0.232 | 0.910 | 0.243 | 278 |
| Perceived barriers to known reporting mechanisms | -0.141 | -1.120 | 0.837 | 0.774 | -0.138 | -1.153 | 0.876 | 0.787 | 91 |

* denotes outcomes where CFS attendance is statistically significant once Bonferroni correction applied; complete case analyses reported only.

**Table S3: Multivariate regressions (adjusted for baseline outcome score, age, gender, school attendance) indicating impact of lower and higher quality CFS attendance at T2 on key outcomes at follow-up**

| **Key Outcomes at Follow-up** | **CFS designated as low quality** | | | | **CFS designated as high quality** | | | | **n** |
| --- | --- | --- | --- | --- | --- | --- | --- | --- | --- |
|  | **Beta** | **95-low** | **95-high** | **p** | **Beta** | **95-low** | **95-high** | **p** |  |
| **Entire cohort** | | | | | | | | | |
| ***Stress*** |  |  |  |  |  |  |  |  |  |
| Child protection concerns | -0.910 | -1.976 | 0.156 | 0.094 | 0.421 | -0.728 | 1.570 | 0.472 | 317 |
| Caregiver stresses | -0.209 | -0.632 | 0.214 | 0.331 | 0.405 | -0.053 | 0.863 | 0.083 | 317 |
| ***Mental Health and Well-being*** |  |  |  |  |  |  |  |  |  |
| Psychosocial well-being | 1.563 | -0.267 | 3.392 | 0.094 | 0.569 | -1.426 | 2.565 | 0.575 | 298 |
| ***Development*** |  |  |  |  |  |  |  |  |  |
| Developmental assests | 2.484 | 0.018 | 4.950 | 0.048 | 1.101 | -1.632 | 3.835 | 0.428 | 248 |
| ***Child protection resources*** |  |  |  |  |  |  |  |  |  |
| Knowledge of CP resources | 0.290 | -0.360 | 0.940 | 0.380 | -0.167 | -0.865 | 0.531 | 0.638 | 276 |
| Perceived barriers to accessing resources | -0.046 | -0.643 | 0.551 | 0.879 | 0.275 | -0.370 | 0.920 | 0.402 | 315 |
| Perceived barriers to known reporting mechanisms | -0.003 | -0.822 | 0.816 | 0.995 | 0.261 | -0.624 | 1.145 | 0.561 | 143 |
| **Girls only** | | | | | | | | | |
| ***Stress*** |  |  |  |  |  |  |  |  |  |
| Child protection concerns | -0.835 | -2.398 | 0.728 | 0.293 | 0.505 | -1.152 | 2.162 | 0.548 | 160 |
| Caregiver stresses | -0.239 | -0.841 | 0.363 | 0.434 | 0.432 | -0.208 | 1.071 | 0.184 | 160 |
| ***Mental Health and Well-being*** |  |  |  |  |  |  |  |  |  |
| Psychosocial well-being | 2.027 | -0.583 | 4.637 | 0.127 | 1.498 | -1.275 | 4.272 | 0.287 | 148 |
| ***Development*** |  |  |  |  |  |  |  |  |  |
| Developmental assests | 2.849 | -0.506 | 6.204 | 0.095 | 2.318 | -1.405 | 6.040 | 0.220 | 122 |
| ***Child protection resources*** |  |  |  |  |  |  |  |  |  |
| Knowledge of CP resources | 0.053 | -0.920 | 1.025 | 0.915 | -0.630 | -1.657 | 0.396 | 0.227 | 142 |
| Perceived barriers to accessing resources | -0.091 | -0.932 | 0.750 | 0.831 | 0.282 | -0.615 | 1.180 | 0.535 | 160 |
| Perceived barriers to known reporting mechanisms | 0.357 | -0.822 | 1.537 | 0.547 | 0.706 | -0.609 | 2.021 | 0.287 | 65 |
| **Boys only** | | | | | | | | | |
| ***Stress*** |  |  |  |  |  |  |  |  |  |
| Child protection concerns | -1.051 | -2.527 | 0.424 | 0.161 | 0.375 | -1.241 | 1.990 | 0.648 | 157 |
| Caregiver stresses | -0.233 | -0.844 | 0.378 | 0.453 | 0.360 | -0.312 | 1.032 | 0.292 | 157 |
| ***Mental Health and Well-being*** |  |  |  |  |  |  |  |  |  |
| Psychosocial well-being | 1.162 | -1.498 | 3.822 | 0.389 | -0.601 | -3.592 | 2.391 | 0.692 | 150 |
| ***Development*** |  |  |  |  |  |  |  |  |  |
| Developmental assests | 2.130 | -1.529 | 5.790 | 0.251 | -0.423 | -4.533 | 3.687 | 0.839 | 126 |
| ***Child protection resources*** |  |  |  |  |  |  |  |  |  |
| Knowledge of CP resources | 0.547 | -0.334 | 1.428 | 0.221 | 0.311 | -0.649 | 1.271 | 0.522 | 134 |
| Perceived barriers to accessing resources | -0.007 | -0.869 | 0.855 | 0.988 | 0.313 | -0.628 | 1.254 | 0.513 | 155 |
| Perceived barriers to known reporting mechanisms | -0.332 | -1.520 | 0.857 | 0.580 | -0.097 | -1.355 | 1.161 | 0.878 | 78 |

* denotes outcomes where CFS attendance is statistically significant once Bonferroni correction applied; complete case analyses reported only.
